# Effect of Proton Pump Inhibitors on *In Vitro* Activity of Tigecycline against Several Common Clinical Pathogens

**DOI:** 10.1371/journal.pone.0086715

**Published:** 2014-01-21

**Authors:** Wentao Ni, Xuejiu Cai, Beibei Liang, Yun Cai, Junchang Cui, Rui Wang

**Affiliations:** 1 Department of Respiratory Diseases, Chinese People’s Liberation Army General Hospital, Beijing, China; 2 Department of Clinical Pharmacology, Chinese People’s Liberation Army General Hospital, Beijing, China; University of Birmingham, United Kingdom

## Abstract

In this study, we evaluated the effect of proton pump inhibitors (PPIs) on *in vitro* antimicrobial activity of tigecycline against several species of clinical pathogens. Clinical non-duplicate isolates of *Acinetobacter baumannii*, *Staphylococcus aureus*, *Enterococcus faecalis* and three species of *Enterobacteriaceae* (*Escherichia coli*, *Klebsiella pneumonia* and *Enterobacter cloacae*) were collected from a tertiary hospital and their MICs of tigecycline alone and in combination with PPIs (omeprazole, lansoprazole and pantoprazole) were determined. With one randomly selected isolate of each bacterial species, an *in vitro* time–kill study was performed for the confirmation of the effect of PPIs on tigecycline activity. The MIC changes after PPIs addition correlated with the PPIs concentrations in the test media. Compared with tigecycline alone, the addition of 5 mg/L PPIs could increase the MICs of tigecycline by 0 to 2-fold and the addition of 50 mg/L PPIs could increase the MICs of tigecycline by 4 to >128-fold. The time–kill study confirmed that the addition of PPIs could affect the *in vitro* activity of tigecycline. Even at low concentration (5 mg/L) of omeprazole and pantoprazole, antagonistic effect could be observed in *E. cloacae* and *E. faecalis* strains. We conclude that *In vitro* activity of tigecycline can be influenced by the presence of PPIs in a concentration-dependent manner.

## Introduction

Tigecycline is the first commercially available member of glycylcyclines which are derived from minocycline. [1] It is a bacteriostatic agent with appealing *in vitro* activity against various multidrug-resistant pathogens such as vancomycin-resistant *Enterococcus faecalis* and *Enterococcus faecium*, methicillin-resistant *Staphylococcus aureus*, *Acinetobacter spp*., and Gram-negative bacteria producing extended-spectrum beta-lactamases. [2] However, since tigecycline has been widely prescribed to treat these organisms, resistant strains are increasingly reported around the world. [3] It's worth noting that Cordina et al. reported the emergence of tigecycline-resistant *E. faecalis* in a patient might be associated with prolonged use of omeprazole. [4] Werner et al. and Yang and Chua showed that addition of omeprazole to test medium could lead to increased MIC of tigecycline in one *E. faecalis* strain and one *A. baumannii* strain respectively. [5], [6] Whether these are accidental phenomena or the concomitant use of omeprazole could influence the activity of tigecycline is worthy of further investigation. And whether other commonly used proton pump inhibitors (PPIs) in clinical practice such as lansoprazole and pantoprazole could also affect the MICs of tigecycline is unknown. Therefore, the present work was done to evaluate the effect of PPIs on *in vitro* antimicrobial activity of tigecycline against several species of clinical pathogens.

## Materials and Methods

### Bacterial Strains

Clinical non-duplicate isolates of *A. baumannii*, *S. aureus*, *E. faecalis* and three species of *Enterobacteriaceae* (*Escherichia coli*, *Klebsiella pneumonia* and *Enterobacter cloacae*) were randomly selected from the specimen database of department of clinical microbiology in our hospital and all strains were collected from hospitalized patients during routine healthy care at different time periods between 2008 and 2011. Data has been de-identified prior to analysis. Most strains were isolated from sputum specimens and all strains were identified using the Vitek II system (bioMe’rieux, Balmes-les-Grottes, France). *E. coli* ATCC 25922 was used as the reference strain.

### Chemicals and Media

Tigecycline was obtained from Wyeth Pharmaceutical (Wyeth Pharmaceutical, Philadelphia, USA). Omeprazole, lansoprazole and pantoprazole standards were purchased from Sigma-Aldrich (Shanghai, China). Mueller Hinton agar (MHA) and Cation-Adjusted Mueller Hinton II Broth (CA-MHB) were purchased from Becton, Dickinson and Co., (Franklin Lakes, NJ, USA). Solutions of all chemicals were freshly prepared on the day of each use, following the manufacturer’s instructions.

### Susceptibility Testing

The *in vitro* antimicrobial susceptibilities for tigecycline alone and in combination with PPIs were determined by agar dilution method. The guidelines and interpretation of the CLSI were followed for the susceptibility determination. [7], [8] In brief, isolates stored at −70°C were thawed, subcultured using MHA plates and incubated for 24 h at 37°C in ambient air. Then, isolated colonies were transferred to CA-MHB and cultures were grown to a cell density of approximately 10^8^ CFU/ml. By using an autoclaved replicator, approximately 10^4^ CFU bacterial cells were inoculated onto MHA plates containing a series of 2-fold concentration increment of tigecycline alone and in combination with either omeprazole (5, 10 or 50 mg/L), lansoprazole (5, 10 or 50 mg/L) or pantoprazole (5, 10 or 50 mg/L). Inoculated MHA plates were incubated at 37°C for 24 h in ambient air. The MIC was defined as the lowest drug concentration that inhibited the visible growth of colonies. All the susceptibility tests were carried out in triplicate on separate days.

### Time-kill Assays

One isolate of each bacterial species was randomly selected for the time-kill assays. Tubes containing freshly prepared CA-MHB supplemented with tigecycline in the presence or absence of PPIs were inoculated with isolates to a density of ∼5×10^5^ CFU/ml in a final volume of 10 ml and incubated in a shaking bath at 37°C. Samples were obtained from each tube at time 0, 3, 6, 12 and 24 h after inoculation and serially diluted in sterile 0.85% sodium chloride solution for determination of viable counts. The Diluted samples, in 0.05-ml aliquots, were plated in duplicate on MHA plates. After the diluted samples incubated at 37°C for 24 h in ambient air, colonies formed were counted, and the total bacterial log_10_ CFU/ml of the original sample was calculated. The concentration of tigecycline used in time-kill assays was 2-fold the MIC value of each isolate that was obtained from the susceptibility testing mentioned in the preceding paragraph. And the concentration of each PPI added in the time-kill assays tubes was 5 mg/L and 50 mg/L. The antagonistic effect of PPIs on tigecycline was interpreted as a ≥2 log_10_ increase in CFU/ml between the combination and tigecycline used alone [9].

## Results and Discussion

Table 1 shows the median value of MICs (MIC_50_) of tigecycline for strains of each species, as a function of adding three kinds of PPIs at different concentrations. There is no change of MICs in all strains with an addition of 5 mg/L lansoprazole and the MICs of 93% strains did not increase with an addition of 5 mg/L omeprazole (data were not shown). However, MIC_50_ values doubled for *E. coli*, *K. pneumoniae* and *E. faecalis* at pantoprazole concentration of 5 mg/L. Omeprazole and pantoprazole at 10 mg/L increased by 2-fold, or 4-fold, the MICs of all species, while the effect of lansoprazole at 10 mg/L was limited to *A. baumannii*. When the concentrations of PPIs added reached to 50 mg/L, MIC values increased substantially. A 4–8 fold increase was seen in lansoprazole, and 32–128 fold or more, increase could be found in omeprazole and pantoprazole.

**Table 1:** Effect of the proton pump inhibitors (PPIs) lansoprazole, omeprazole and pantoprazole at three different concentrations on the MICs of tigecycline in clinical isolates of 6 species of pathogens.

Species	n^a^	MIC_50_ (mg/L)									
		Tigecyclinealone	+ Lansoprazole			+ Omeprazole			+ Pantoprazole		
			5 mg/L	10 mg/L	50 mg/L	5 mg/L	10 mg/L	50 mg/L	5 mg/L	10 mg/L	50 mg/L
*E. coli*	12	0.25	0.25	0.25	**2**	0.25	**0.5**	**32**	**0.5**	**0.5**	>**32**
*K. pneumonia*	10	0.5	0.5	0.5	**4**	0.5	**1**	**32**	**1**	**2**	>**32**
*E. cloacae*	12	0.5	0.5	0.5	**4**	0.5	**1**	**32**	0.5	**1**	>**32**
*A. baumannii*	12	0.5	0.5	**1**	**4**	0.5	**1**	**16**	0.5	**1**	**16**
*S. aureus*	12	0.25	0.25	0.25	**1**	0.25	**0.5**	**8**	0.25	**0.5**	**8**
*E. faecalis*	10	0.125	0.125	0.125	**1**	0.125	**0.25**	**8**	**0.25**	**0.5**	**16**

a The number of strains of each species tested in the study.

Increased MICs in >50% of isolates are indicated in boldface.

**Figure 1:**
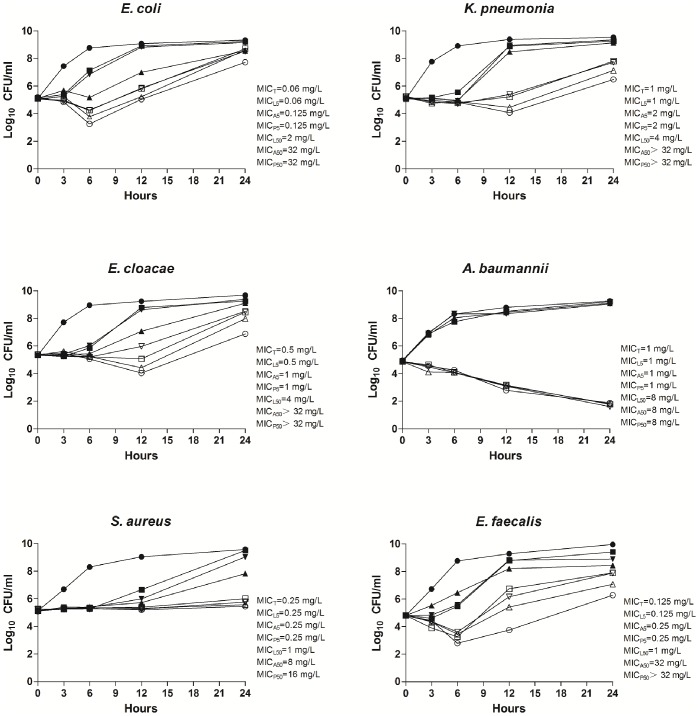
Time–kill curves showing effects of proton pump inhibitors (PPIs) on the activity of tigecycline. MIC_T_, MIC for tigecycline alone; MIC_L5_ and MIC_L50_, MIC for tigecycline in combination with 5 mg/L and 50 mg/L lansoprazole; MIC_A5_ and MIC_A50_, MIC for tigecycline in combination with 5 mg/L and 50 mg/L omeprazole; MIC_P5_ and MIC_P50_, MIC for tigecycline in combination with 5 mg/L and 50 mg/L pantoprazole. •, Control; ▴, 50 mg/L lansoprazole; ▾, 50 mg/L omeprazole; ▪, 50 mg/L pantoprazole; Δ, 5 mg/L lansoprazole; ▽, 5 mg/L omeprazole; □, 5 mg/L pantoprazole; ○, tigecycline alone. The *in vitro* time-kill experiments were duplicated; mean values are plotted. In all duplicate experiments, similar time-kill results were obtained.

To confirm the effect of PPIs on tigecycline activity, we performed the time-kill assays of tigecycline for one randomly selected isolate of each species, in the presence or absence of PPIs at two different concentrations (50 and 5 mg/L). As displayed in Figure 1, time-kill data demonstrated antagonistic effect for all PPIs at high concentration (50 mg/L). The antagonistic effect was observed at 3 h for *A. baumannii*, at 6 h for *E. coli* and *E. faecalis*, at 12 h for *K. pneumonia* and *E. cloacae*, and at 24 h for *S. aureus*. For most of the time, bacterial colony counts in lansoprazole (50 mg/L) group was lower than in omeprazole (50 mg/L) and pantoprazole (50 mg/L) group, except for the *A. baumannii* strain which has the same MIC value for the three PPIs. With the addition of omeprazole and pantoprazole at 5 mg/L, bacterial colony counts of four strains (*E. coli*, *K. pneumonia*, *E. cloacae* and *E. faecalis*) were increased by at least 1 log_10_ CFU/mL when compared with tigecycline alone at 12 h. The antagonistic effect could be observed in *E. cloacae* at 12 h for pantoprazole and at 24 h for omeprazole. And at 12 h, antagonistic effect could also be found in *E. faecalis* in the presence of 5 mg/L omeprazole or pantoprazole.

These data show that *in vitro* susceptibility of tigecycline can be influenced by an addition of PPIs in the test medium. The effect of PPIs seems negligible for most clinical strains when the concentration is low except for pantoprazole which increased the MIC_50_ for *E. coli*, *K. pneumoniae* and *E. faecalis* at the concentration of 5 mg/L. Understanding the reason for such liquidity spillovers is of broad interest because it can be clarifying on sudden and short systematic liquidity crises. Nonetheless, the liquidity spillover causes are not yet well understood. But with much higher doses of 50 mg/L, the susceptibility decreased dramatically, indicating the influence might be concentration-dependent. In addition, the effect of PPIs on the activity of tigecycline may differ among different species of pathogens. For example, after addition of 50 mg/L omeprazole, the MIC_50_ of *E. coli* increased by 128-fold while the MIC_50_ of *S. aureus* increased by 32-fold, as shown in Table 1.

The influence of PPIs on *in vitro* susceptibilities of tigecycline suggests that the concomitant use of PPIs may weaken its antibacterial activity in the clinic. After intravenous administration of lansoprazole 30 mg twice daily, the C_max_ in plasma among healthy volunteers were approximately 1.45–2.2 mg/L. [10] And after intravenous administration of omeprazole 40 mg every 24 h, the mean C_max_ in plasma were 2.51±0.65 mg/L in individuals with homozygous extensive metabolizer and 3.45±0.65 mg/L in those with poor metabolizer. [11] In our study, we found that an addition of 5 mg/L lansoprazole or omeprazole had no or little effects on the susceptibility of tigecycline, which implies that the use of these two PPIs at routine dosage regimen may not influence the antimicrobial activity of tigecycline. However, following single intravenous infusion of pantoprazole at a dose of 40 mg administered over 15 min, the C_max_ in serum ranged from 3.21–7.05 mg/L in healthy male subjects. [12] And the MIC_50_ values doubled for *E. coli*, *K. pneumoniae* and *E. faecalis* at pantoprazole concentration of 5 mg/L. This suggests that the activity of tigecycline against some pathogens may be affected by the concomitant use of pantoprazole under physiological conditions.

The mechanism by which PPIs influence *in vitro* activity of tigecycline is still unclear. This influence appears to be specific to tigecycline, because PPIs did not increase the MICs of other antibiotics, such as tetracycline, meropenem, ceftazidime, levofloxacin, gentamicin and streptomycin. [6], [13] As effective agents inactivate H+,K+ ATPase in human parietal cells, [14] PPIs may also play a role on the H+,K+ ATPase in bacterial cells and then affect uptake of the drug. Additionally, except for inhibiting proton pumps, the toxicity of PPIs may impair other efflux pumps of bacterial cells, which may increase the MICs for those bacteria as well.

In conclusion, the findings of this study demonstrate PPIs can influence *in vitro* antibacterial activity of tigecycline in a concentration-dependent manner. Compared with lansoprazole and omeprazole, pantoprazole is more likely to interfere with the antimicrobial activity of tigecycline in the clinic when human body pharmacokinetics of these PPIs was considered. Since drug concentrations tested in our study were static and the elimination half-life of PPIs in serum is shorter than that of tigycycline, further *in vivo* studies using ideally animal models are needed to confirm these conclusions.
